# Supplementary material for: Biogenic Synthesis of Copper Nanoparticles Using Bacterial Strains Isolated from an Antarctic Consortium Associated to a Psychrophilic Marine Ciliate: Characterization and Potential Application as Antimicrobial Agents
Source: Mar Drugs. 2021 May 8;19(5):263. doi: 10.3390/md19050263 (PMC8151786; doi:10.3390/md19050263)
Supplement: Supplementary file 1 [file marinedrugs-19-00263-s001.zip › marinedrugs-1186236-supplementary.pdf]

Biogenic Synthesis of Copper Nanoparticles Using Bacterial Strains Isolated from an Antarctic Consortium Associated to a Psychrophilic Marine Ciliate: Characterization and Potential Application as Antimicrobial Agents

Maria Sindhura John [1], Joseph Amruthraj Nagoth [1], Marco Zannotti [2,*], Rita Giovannetti [2], Alessio Mancini [1], Kesava Priyan Ramasamy [1], Cristina Miceli [1] and Sandra Pucciarelli [1,*]

Supplementary figures

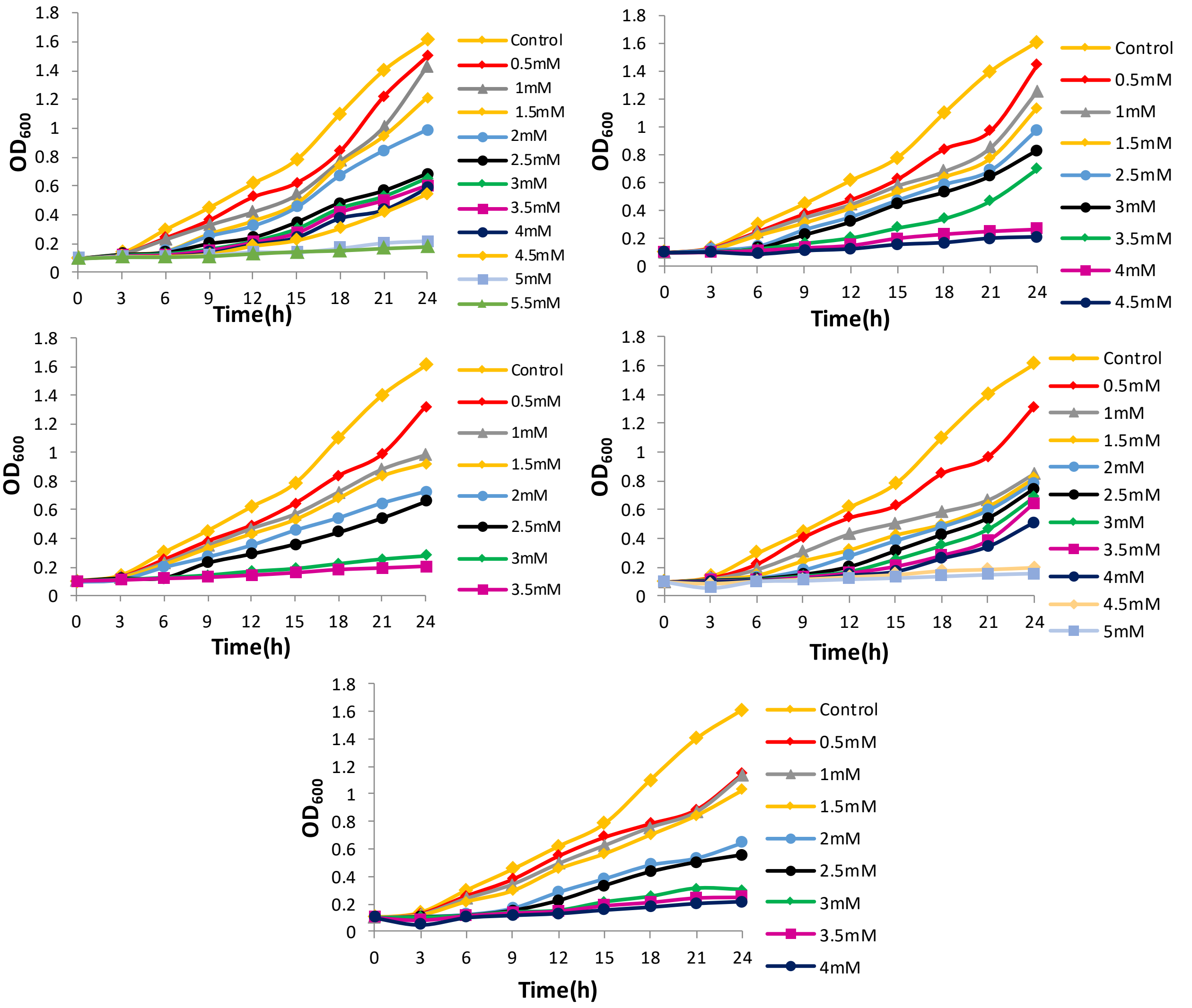

Figure S1. Bacterial growth in the presence of increasing concentration of $CuSO_4$.

A B C

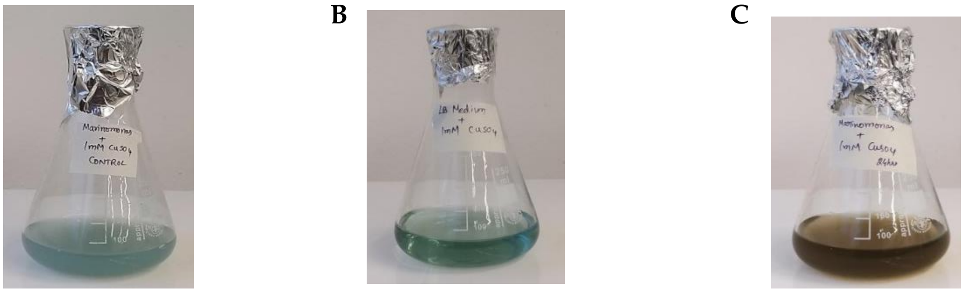

Marinomonas ef1

A B C

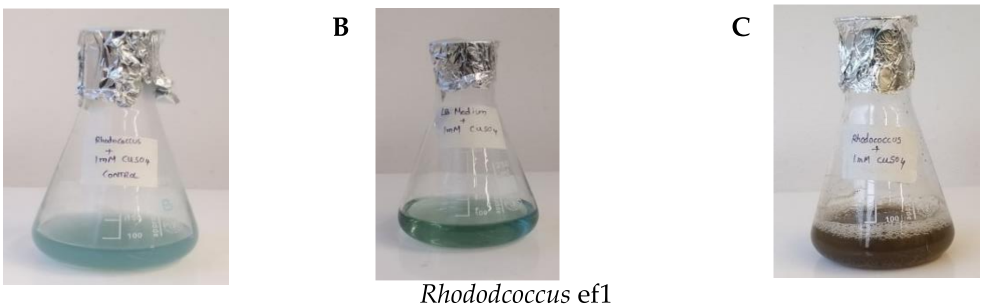

Rhododcoccus ef1

A B C

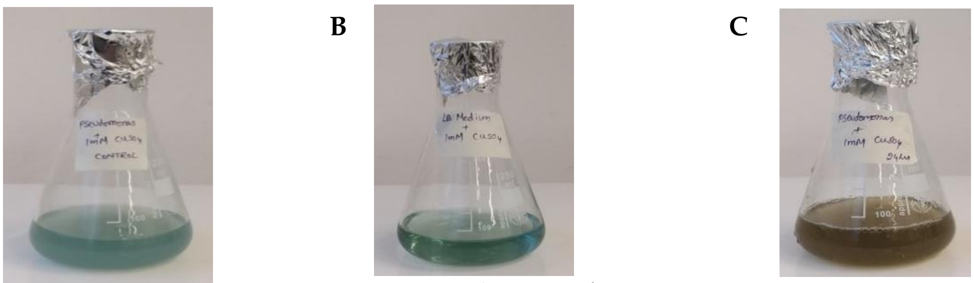

Pseudomonas ef1

A B C

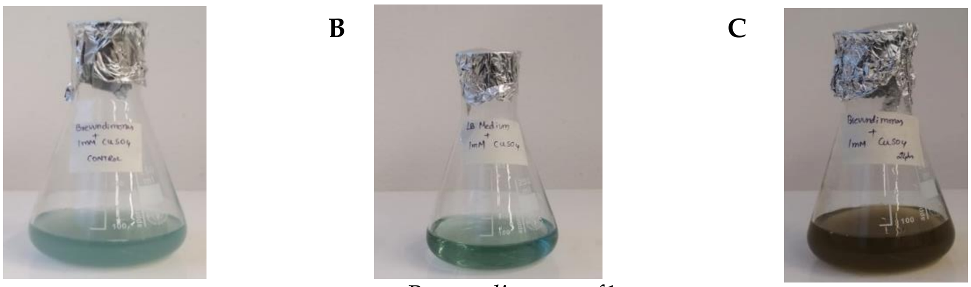

Brevundimonas ef1

A B C

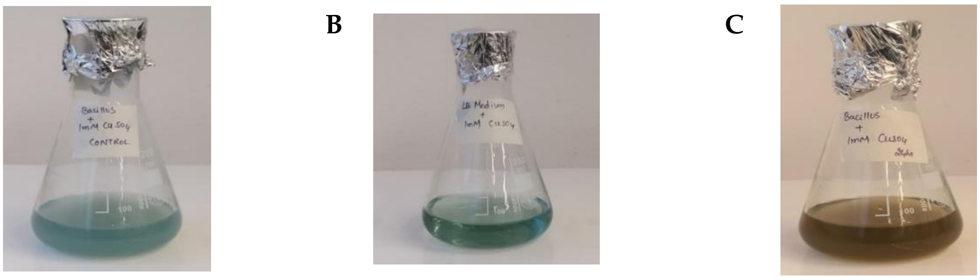

Bacillus ef1

Figure. S2. Biosynthesis of Cu NPs from *Marinomonas* ef1, *Rhododcoccus* ef1, *Pseudomonas* ef1, *Brevundimonas* ef1 and *Bacillus* ef1. **A**. Control with heat killed bacterial culture with 1mM CuSO4; **B**. Control with LB medium with 1mM CuSO4; **C**. Biosynthesized Cu NPs.

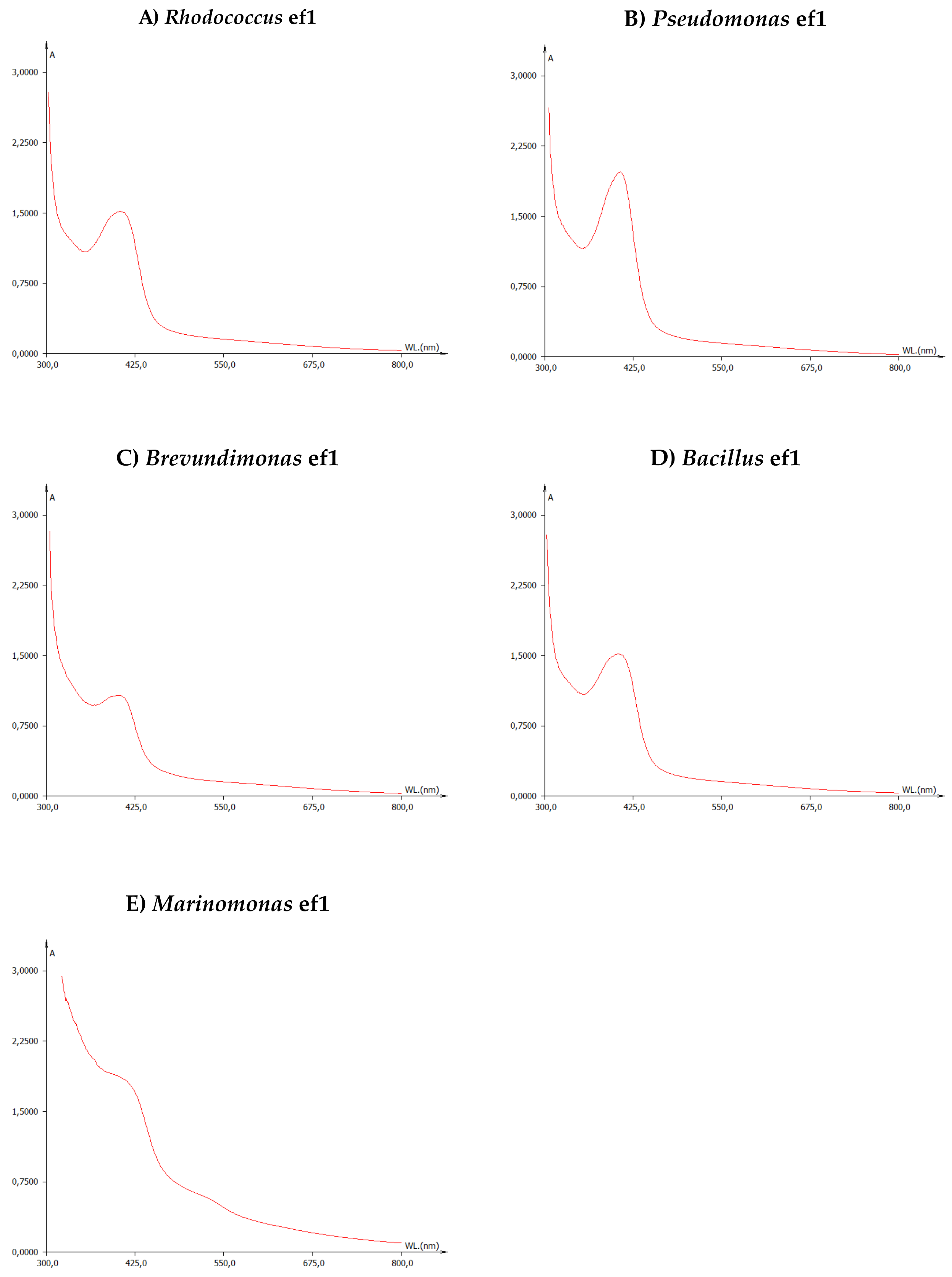

Figure S3. UV−Vis absorbance spectra of bio- CuO NPs synthesized from (**A**) *Rhodococcus* ef1, (**B**) *Pseudomonas* ef1, (**C**) *Brevundimonas* ef1; **D**) *Bacillus* ef1; (**E**) *Marinomonas* ef1.

IR spectra. All the samples showed similar IR spectra with comparable vibration peaks. In the IR spectra a broad intense absorption band in the region between 3600 and 2800 cm^{-1} can be attributed to –OH stretching, the peak at around 3245 cm^{-1} can be attributed to −NH of amide. The band at around 3065 cm^{-1} represents the =C–H stretch of aromatic and unsaturated hydrocarbons; on the other hand the band at around 2960 cm^{-1} is due to the C–H stretching of the aliphatic portion of the molecule around nanoparticles. In the range between 2100 to 1800 cm^{-1} the bands can be assigned to the aromatic portion inside the biomolecules. At around 1620–1650 it is possible to observe the carbonyl group C=O of amide and also in this region can be possible the observe the bending of the unsaturated C=C bending; in addition, the band at around 1550 cm^{-1} is representative of the N–H bending of the secondary amide. The peak at around 1440–1455 cm^{-1} indicates the bending of the aliphatic –C–H and –CH_3. The band at around 1390 cm^{-1} can be attributed to the –OH bending. The peak at around 1240 cm^{-1} can be assigned to C–N stretching vibration of amide, and the peak at around 1100 cm^{-1} to the C–O stretching of alcohol. The broad adsorption at around 900–800 cm^{-1} can be due to the rocking vibration out of plane of –NH portion of the amide.

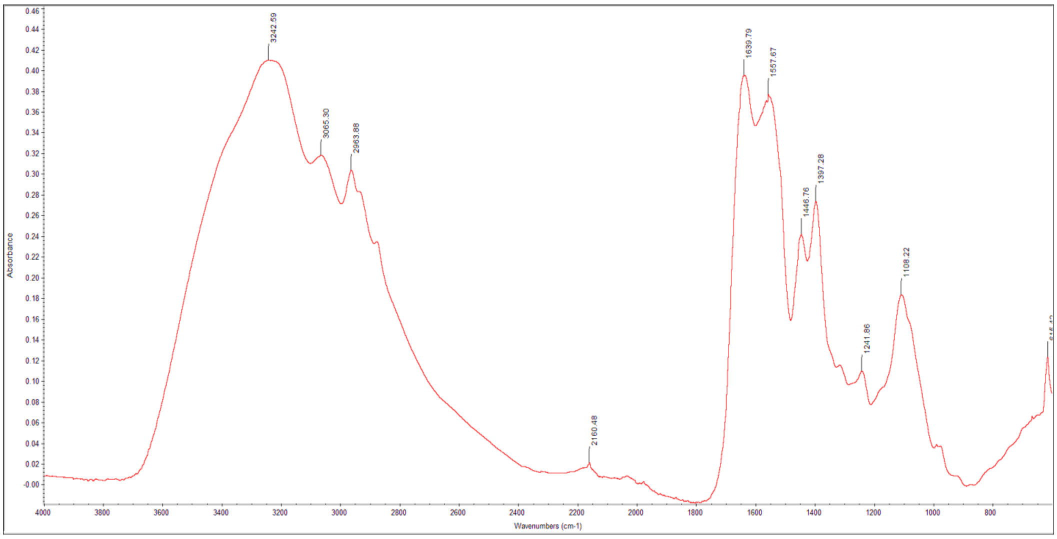

IR Absorption (cm^{-1})	Band Assignments
3242.59	–NH stretch
3065.30	=C–H stretch of aromatic and unsaturated hydrocarbons
2963.88	–CH stretch of aliphatic compounds
1639.79	–C=O stretching amide, bending C=C
1557.67	–NH in plane bend amide
1446.76	–C–H and –CH_3 in Aliphatic -compounds
1397.28	–OH bending
1241	C–N stretch amide
1108.82	C–OH stretching

Figure S4. FTIR spectrum of biosynthesized CuO NPs from *Marinomonas* ef1 and IR assignments.

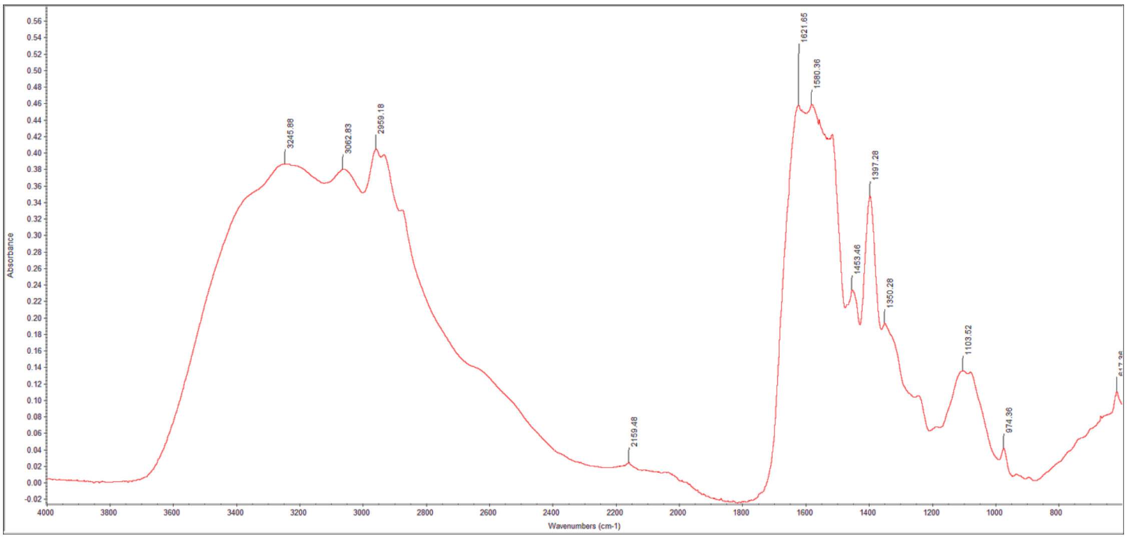

IR Absorption (cm⁻¹)	Band Assignments
3245.88	–NH stretch
3062.83	=C–H stretch of aromatic and unsaturated hydrocarbons
2959.18	–CH stretch of aliphatic compounds
1621.65	–C=O stretching amide, bending C=C
1580.36	–NH in plane bend amide
1453.46	–C–H and –CH_3 in Aliphatic -compounds
1397.28	–OH bending
1240	C–N stretch amide
1103.52	C–OH stretching

Figure S5. FTIR spectrum of biosynthesized CuO NPs from *Rhodococcus* ef1 and IR assignments.

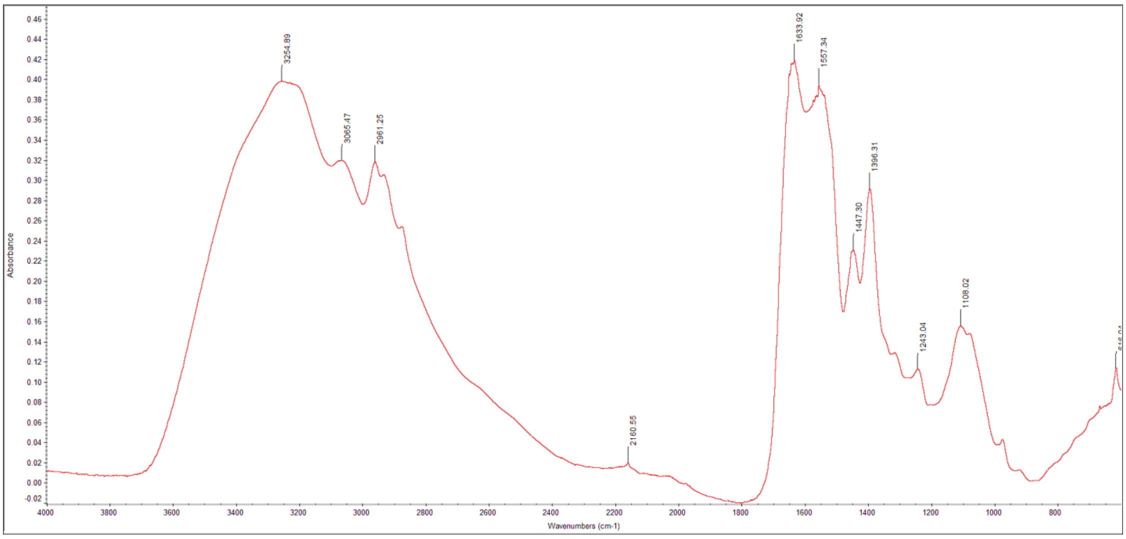

IR Absorption (cm⁻¹)	Band Assignments
3254.89	–NH stretch
3065.47	=C–H stretch of aromatic and unsaturated hydrocarbons
2961.25	–CH stretch of aliphatic compounds
1633.92	–C=O stretching amide, bending C=C
1557.34	–NH in plane bend amide
1447.30	–C–H and –CH_3 in Aliphatic -compounds
1396.31	–OH bending
1243.04	C–N stretch amide
1108.02	C–OH stretching

Figure S6. FTIR spectrum of biosynthesized CuO NPs from *Pseudomonas* ef1 and IR assignments.

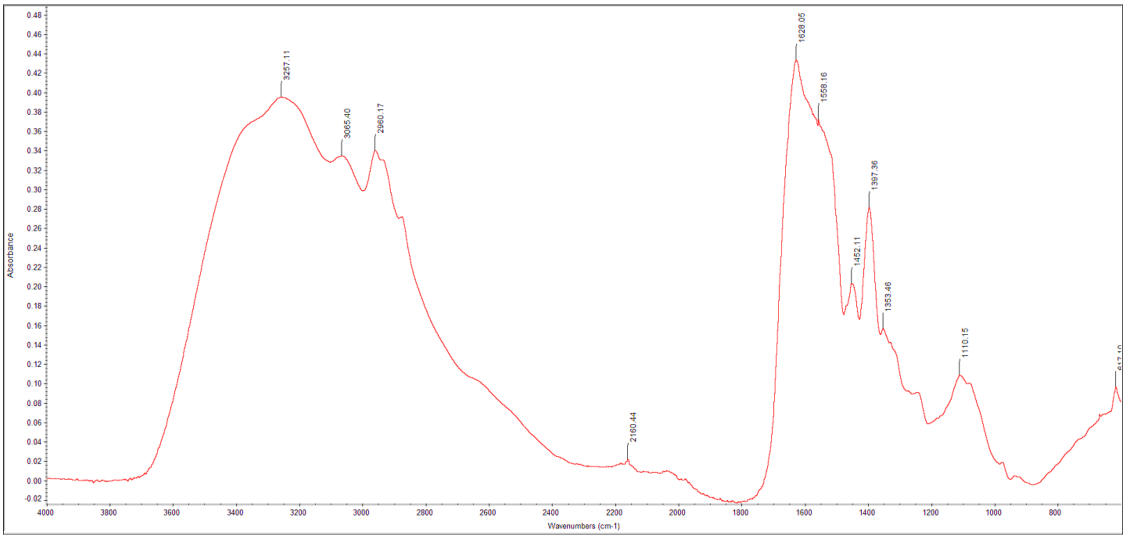

IR Absorption (cm^{-1})	Band Assignments
3257.11	–NH stretch
3065.40	=C–H stretch of aromatic and unsaturated hydrocarbons
2960.70	–CH stretch of aliphatic compounds
1628.01	–C=O stretching amide, bending C=C
1558.16	-NH in plane bend amide
1452.11	–C–H and –CH_3 in Aliphatic -compounds
1397.36	–OH bending
1240.21	C–N stretch amide
1110.15	C–OH stretching

Figure S7. FTIR spectrum of biosynthesized CuONPs from *Brevundimonas* ef1 and IR assignments.

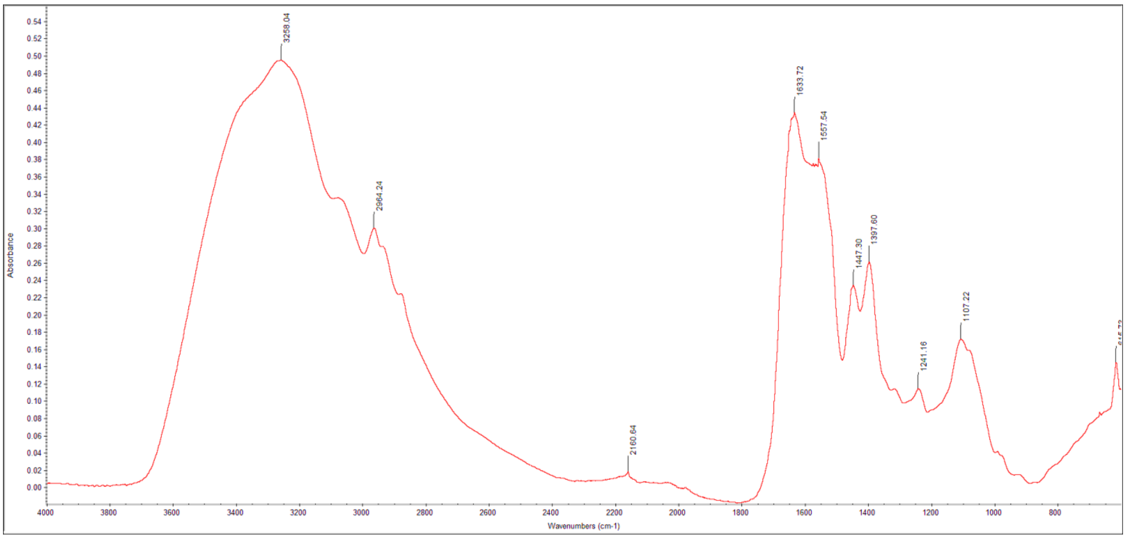

IR Absorption (cm^{-1})	Band Assignments
3258.04	–NH stretch
3064.25	=C–H stretch of aromatic and unsaturated hydrocarbons
2964.24	–CH stretch of aliphatic compounds
1633.72	–C=O stretching amide, bending C=C
1557.54	–NH in plane bend amide
1447.30	–C–H and –CH_3 in Aliphatic -compounds
1397.60	–OH bending
1241.16	C–OH stretching

Figure S8. FTIR spectrum of biosynthesized CuO NPs from *Bacillus* ef1 and IR assignments.

Figure S9. MIC values of *Marinomonas* CuO NPs. Among Gram-negative bacteria *Proteus mirabilis* showed the lowest MIC of 3.12 μg/mL whereas *Pseudomonas sp., Citrobacter koseri* and *Serratia marcescens* showed a MIC of 6.25 μg/mL. While *Escherichia coli, Klebsiella pneumoniae* and *Acinetobacter baumanii* showed a MIC value of 12.5 μg/mL. In gram positive bacteria *Staphylococcus aureus* showed 12.5 μg/mL. Among fungi the lowest MIC value was in *Candida parapsilosis* (12.5 μg/mL), while *Candida albicans* showed a MIC of 25 μg/mL. The lowest MBC among gram negative bacteria was showed by *Proteus mirabilis* and *Citrobacter koseri* is 6.25 μg/mL. *Pseudomonas sp., Acinetobacter baumanii* and *Serratia marcescens* showed a MBC of 12.5 μg/mL. Among gram positive bacteria *Staphylococcus aureus* showed a MBC of 25 μg/mL.Among Fungi *Candida albicans* and *Candida parapsilosis* showed MBC value of 25 μg/mL.

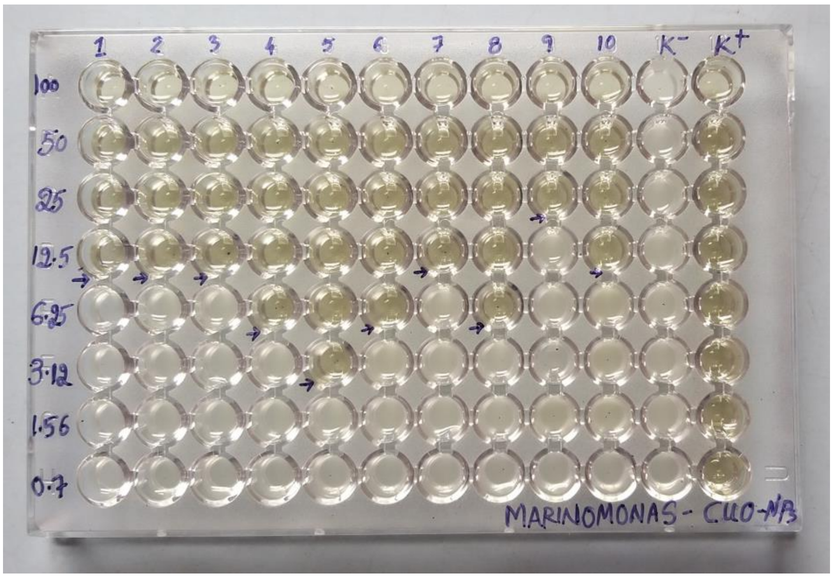

Marinomonas ef1 CuO NPs Broth microdilution method. 1. *Staphylococcus aureus* 2. *Escherichia coli* 3. *Klebsiella pneumoniae* 4. *Pseudomonas sp.* 5. *Proteus mirabilis* 6. *Citrobacter koseri* 7. *Acinetobacter baumanii* 8. *Serratia marcescens* 9. *Candida albicans* 10. *Candida parapsilosis.*

Figure S10. MIC values of *Rhodococcus* ef1 CuO NPs. Among Gram-negative bacteria *Serratia marcescens* showed the lowest MIC (3.12 µg/mL) whereas *Klebsiella pneumoniae* and *Proteus mirabilis* showed a MIC of 6.25 µg/mL. *Escherichia coli*, *Pseudomonas sp.*, *Citrobacter koseri* and *Acinetobacter baumanii* showed a MIC value of 12.5 µg/mL. In gram-positive bacteria, *Staphylococcus aureus* showed a MIC of 12.5 µg/mL. The lowest MIC in fungi was in *Candida albicans* (12.5 µg/mL). *Candida parapsilosis* showed a MIC of 25 µg/mL. The lowest MBC among gram-negative bacteria was showed by *Klebsiella pneumoniae*, *Proteus mirabilis*, *Citrobacter koseri*, *Acinetobacter baumanii* and *Serratia marcescens* (12.5 µg/mL). *Escherichia coli* and *Pseudomonas sp.* showed a MBC of 25 µg/mL. Among gram-positive bacteria *Staphylococcus aureus* showed a MBC of 25 µg/mL. *Candida albicans* and *Candida parapsilosis also* showed a MBC of 25 µg/mL.

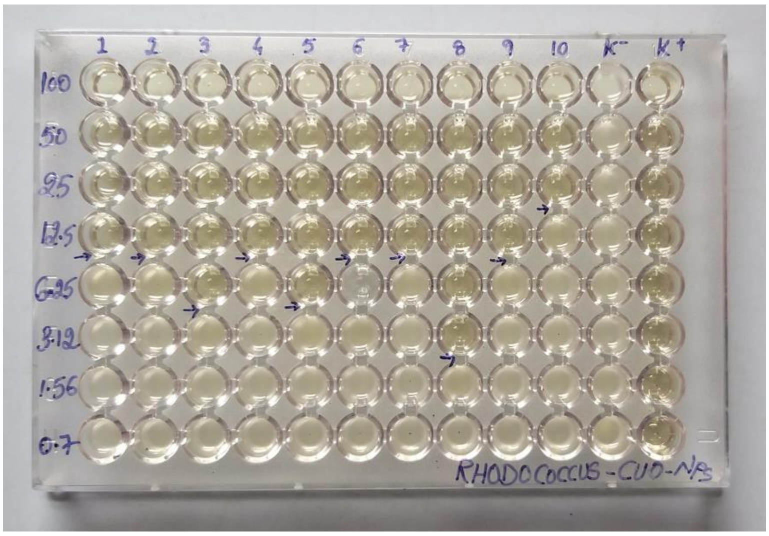

Rhodococcus ef1 CuO NPs Broth microdilution method. 1. *Staphylococcus aureus* 2. *Escherichia coli* 3. *Klebsiella pneumoniae* 4. *Pseudomonas sp.* 5. *Proteus mirabilis* 6. *Citrobacter koseri* 7. *Acinetobacter baumanii* 8. *Serratia marcescens* 9. *Candida albicans* 10. Candida parapsilosis.

Figure S11. MIC values of *Brevundimonas* ef1 CuO NPs. *Proteus mirabilis* and *Serratia marcescens* showed the lowest MIC (6.25 µg/mL), whereas *Klebsiella pneumoniae*, *Pseudomonas sp.*, *Citrobacter koseri* and *Acinetobacter baumanii* showed a MIC value of 12.5 µg/mL. *Escherichia coli* showed a MIC value of 25 µg/mL. In gram-positive bacteria, *Staphylococcus aureus* showed a MIC value of 25 µg/mL. Among fungi, *Candida albicans* and *Candida parapsilosis* showed a MIC value of 12.5 µg/mL. The lowest MBC value among gram negative bacteria was by *Proteus mirabilis* and *Serratia marcescens* (12.5 µg/mL). *Escherichia coli*, *Klebsiella pneumoniae*, *Pseudomonas sp.*, *Citrobacter koseri* and *Acinetobacter baumanii* showed a MBC value of 25 µg/mL. Among gram positive bacteria *Staphylococcus aureus* showed a MBC of 25 µg/mL. *Candida albicans* and *Candida parapsilosis* showed a MBC value of 25 µg/mL.

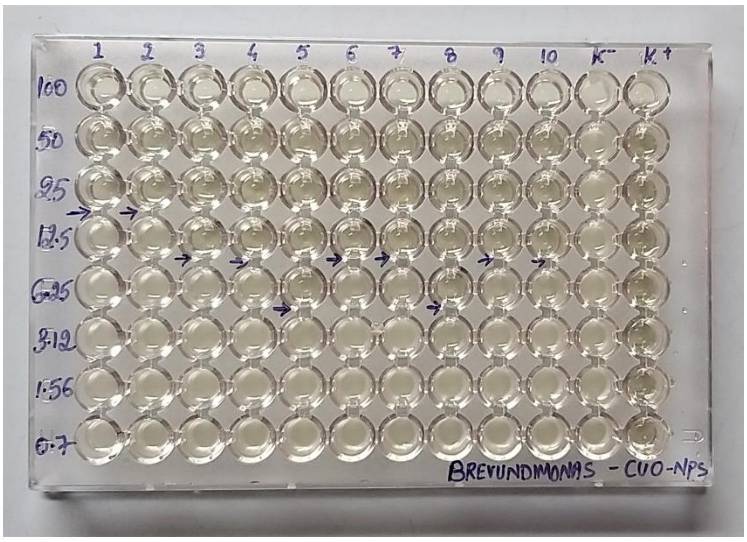

Brevundimonas ef1 CuO NPs Broth microdilution method. 1. *Staphylococcus aureus* 2. *Escherichia coli* 3. *Klebsiella pneumoniae* 4. *Pseudomonas sp.* 5. *Proteus mirabilis* 6. *Citrobacter koseri* 7. *Acinetobacter baumanii* 8. *Serratia marcescens* 9. *Candida albicans* 10. Candida parapsilosis.

Figure S12. MIC values of *Pseudomonas* ef1 CuO NPs. Proteus mirabilis, *Citrobacter koseri* and *Serratia marcescens* showed the lowest MIC (6.25 µg/mL). *Klebsiella pneumoniae*, *Pseudomonas sp.*, and *Acinetobacter baumanii* showed a MIC value of 12.5 µg/mL. *Escherichia coli* showed a MIC value of 25 µg/mL. In gram positive bacteria *Staphylococcus aureus* showed a MIC value of 12.5 µg/mL. Among fungi *Candida albicans* and *Candida parapsilosis* showed MIC value of 25 µg/mL and 12.5 µg/mL respectively. The Lowest MBC was in *Pseudomonas sp.*, *Proteus mirabilis*, *Citrobacter koseri*, *Acinetobacter baumanii* and *Serratia marcescens* (12.5 µg/mL). *Escherichia coli* and *Klebsiella pneumoniae* showed a MBC value of 25 µg/mL. Among gram positive bacteria *Staphylococcus aureus* showed a MBC of 25 µg/mL. *Candida albicans* and *Candida parapsilosis* showed a MBC value of 25 µg/mL.

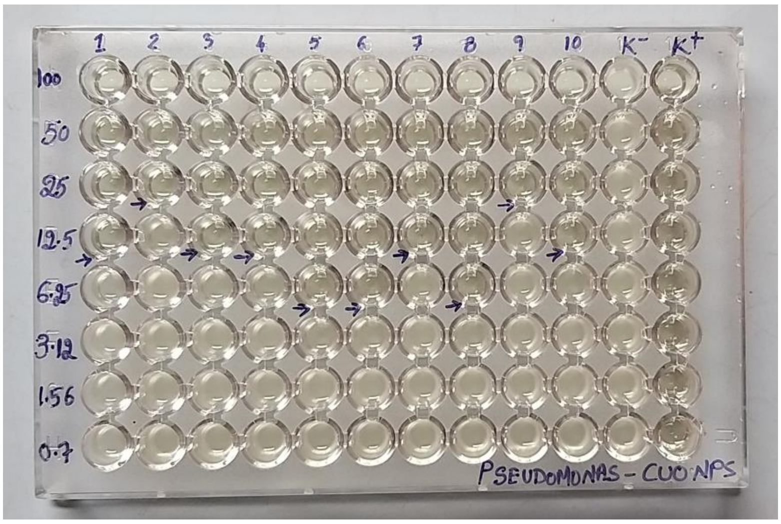

Pseudomonas ef1 CuO NPs Broth microdilution method. 1. *Staphylococcus aureus* 2. *Escherichia coli* 3. *Klebsiella pneumoniae* 4. *Pseudomonas sp.* 5. *Proteus mirabilis* 6. *Citrobacter koseri* 7. *Acinetobacter baumanii* 8. *Serratia marcescens* 9. *Candida albicans* 10. Candida parapsilosis.

Figure S13. MIC values of *Bacillus* ef1 CuO NPs. *Klebsiella pneumoniae*, *Proteus mirabilis* and *Serratia marcescens* showed the lowest MIC (6.25 μg/mL), whereas *Escherichia coli*, *Pseudomonas sp.*, *Citrobacter koseri* and *Acinetobacter baumanii* showed a MIC of 12.5 μg/mL. *Escherichia coli* showed a MIC of 25 μg/mL. In gram positive bacteria *Staphylococcus aureus* showed a MIC value of 12.5 μg/mL. Among fungi *Candida albicans* and *Candida parapsilosis* showed a MIC of 12.5 μg/mL and 6.25 μg/mL respectively. The lowest MBC value among gram-negative bacteria was in *Klebsiella pneumoniae* (6.25 μg/mL). *Escherichia coli*, *Pseudomonas sp.*, *Proteus mirabilis*, *Citrobacter koseri*, *Acinetobacter baumanii* and *Serratia marcescens* showed a MBC of 12.5 μg/mL. Among gram positive bacteria *Staphylococcus aureus* showed a MBC of 12.5 μg/mL. Among Fungi *Candida albicans* and *Candida parapsilosis* showed MBC of 25 μg/mL and 12.5 μg/mL respectively.

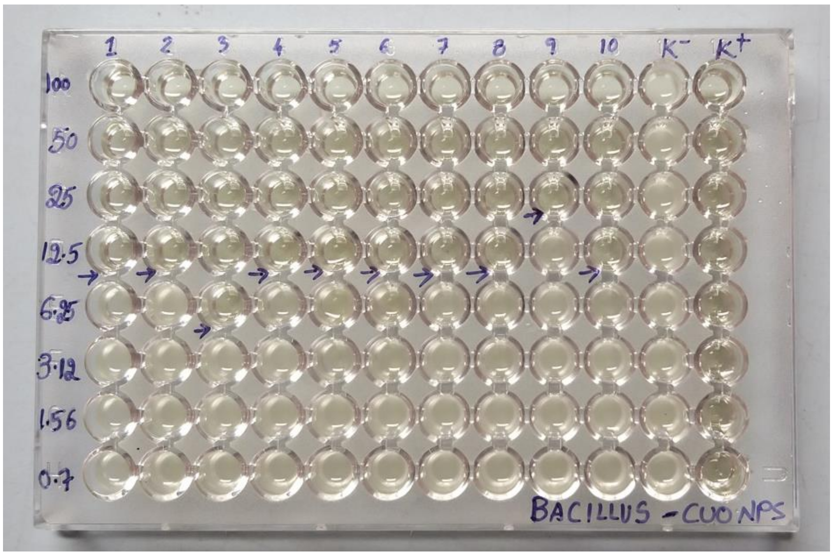

Bacillus ef1 CuO NPs Broth microdilution method .1. *Staphylococcus aureus* 2. *Escherichia coli* 3. *Klebsiella pneumoniae* 4. *Pseudomonas sp.* 5. *Proteus mirabilis* 6. *Citrobacter koseri* 7. *Acinetobacter baumanii* 8. *Serratia marcescens* 9. *Candida albicans* 10. Candida parapsilosis.
